# Caudal-Threaded Versus Direct Lumbar/Thoracic Epidural Catheters in Small-Weight Infants—A Single-Center Retrospective Observational Study

**DOI:** 10.3390/children13091148

**Published:** 2026-08-26

**Authors:** Mihaela Visoiu, Tyler H. Augi, Franklyn Paul Cladis

**Affiliations:** 1 Department of Anesthesiology and Perioperative Medicine, UPMC Children’s Hospital of Pittsburgh, School of Medicine, University of Pittsburgh, Pittsburgh, PA 15224, USA; cladfp@upmc.edu; 2 School of Medicine, University of Pittsburgh, A1305 Scaife Hall, 3550 Terrace Street, Pittsburgh, PA 15213, USA; tha25@pitt.edu

**Keywords:** caudal-threaded epidural catheter, direct lumbar/thoracic epidural catheter, small weight infants, successful placement, complication

## Abstract

**Highlights:**

**What are the main findings?**

- Epidural catheters were successfully placed in 96.2% of small-weight infants (median weight 3 kg), and caudal-threaded and direct lumbar/thoracic techniques produced no statistically significant between-group differences of postoperative pain and sedation scores and opioid consumption.
- Compared with the caudal-threaded approach, direct lumbar/thoracic epidural placement required longer procedure times and was associated with a higher incidence of catheter-related complications (20% vs. 4%; *p* = 0.02), primarily catheter leakage requiring premature catheter removal; however, given differences in epidural management between groups, these complications cannot be attributed to the catheter insertion site alone.

**What is the implication of the main finding?**

- Both techniques are effective options for postoperative analgesia in this population when managed by a dedicated Pediatric Acute Pain Service so that technique selection can be guided by the clinical scenario and required level of segmental analgesia.
- The higher risk of leakage and premature removal with direct lumbar/thoracic catheters should be weighed against its benefits when clinicians choose an epidural approach for small-weight infants.

**Abstract:**

**Background**: Continuous epidural analgesia is widely used for postoperative pain management in small infants. Epidural catheters may be inserted via a caudal-threaded approach or by direct lumbar/thoracic placement; however, comparative data on placement success, clinical use, postoperative analgesic outcomes, and complications remain limited. This study compared these two epidural techniques in small-weight infants undergoing surgery. **Methods:** This retrospective observational cohort study included all infants admitted to the Neonatal Intensive Care Unit at UPMC Children’s Hospital of Pittsburgh with documented epidural catheter placement in the chart between January 2018 and December 2024. Demographic and perioperative data were extracted from the electronic medical record. Outcomes included technical success of catheter placement; patient, surgical, and epidural characteristics; epidural medications; postoperative pain and sedation scores; opioid consumption; and epidural catheter complications. **Results**: A total of 104 patients had documentation of 105 epidural catheter placements in the chart; five procedures were unsuccessful in four patients, resulting in 100 successful epidural catheter placements (70 caudal-threaded and 30 direct lumbar/thoracic). Patient and surgical characteristics, postoperative pain and sedation scores, and opioid consumption did not differ significantly between groups. Direct lumbar/thoracic epidural catheter placement required longer procedure times. Patients in the direct lumbar/thoracic epidural group were more likely than those in the caudal-threaded group to receive a ropivacaine/clonidine infusion (47% vs. 4%; *p* < 0.001) and a programmed intermittent bolus of epidural infusion (60% vs. 3%; *p* < 0.001). The direct lumbar/thoracic group experienced more postoperative catheter-related complications than the caudal-threaded group (20% vs. 4%; *p* = 0.02), primarily catheter leakage requiring premature catheter removal. **Conclusions:** Epidural catheter placement was successful in 96.2% of patients. No statistically significant between-group differences were detected in postoperative pain scores, sedation scores, or opioid consumption between caudal-threaded and direct lumbar/thoracic epidural catheters in small-weight infants (median 3 kg) when placed by a Pediatric Acute Pain Service. Direct lumbar/thoracic epidural catheters were associated with a higher incidence of postoperative catheter leakage and premature catheter removal; however, these complications cannot be attributed solely to the location of epidural catheter insertion. These findings may help clinicians select the most appropriate epidural technique for postoperative pain management in this population.

## 1. Introduction

Continuous epidural analgesia via a caudal or lumbar/thoracic approach is an established technique for providing perioperative analgesia in small-weight infants [1,2,3,4,5,6,7,8,9,10]. In these patients, the relative fluidity of epidural fat permits caudal epidural catheters to be advanced cranially to the lumbar or thoracic levels [11,12]. This approach is often used because it is technically easier, and ultrasound guidance facilitates needle placement through the sacral hiatus and confirms catheter location within the epidural space at the desired spinal level [13,14]. Unfortunately, advancing the catheter from the sacral canal over several vertebral levels may result in catheter misplacement or failure to reach the intended spinal level. Direct lumbar or thoracic epidural catheter placement offers an alternative by allowing insertion at or near the desired dermatomal level [15]. This technique may be advantageous for procedures requiring precise segmental analgesia in small infants undergoing surgery. However, it is technically more demanding and is generally performed by clinicians with expertise in pediatric regional anesthesia. Although both caudal-threaded and direct lumbar/thoracic epidural catheter techniques are routinely used in small infants, comparative data on their clinical applications and outcomes remain limited. Little is known about the patient populations selected for each technique, the surgical procedures for which each approach is used, epidural catheter characteristics, the technical success of catheter placement, postoperative analgesic outcomes, and epidural catheter-related complications. Consequently, the choice of epidural approach is largely based on clinician experience and institutional practice rather than comparative clinical evidence.

This study describes one institution’s experience with caudal-threaded and direct lumbar/thoracic epidural catheters placed in infants admitted after surgery to the Neonatal Intensive Care Unit (NICU). The patients’ median weight was 3 kg. It focuses on the clinical applications of both techniques, including patient characteristics, surgical procedures, epidural catheter insertion location, and medications used for epidural analgesia. The primary objective was to describe the technical success of each procedure. Secondary objectives were to compare postoperative analgesic outcomes, including postoperative pain and sedation scores, opioid consumption, and adjunct analgesic use, and to compare the incidence of intraoperative and postoperative complications between the two techniques. We hypothesized that direct lumbar/thoracic epidural catheter placement would provide postoperative analgesia comparable to that of caudal-threaded epidural catheters. Still, we would differ in procedural characteristics and catheter-related placement challenges and complications.

## 2. Materials and Methods

### 2.1. Study Design

This retrospective observational cohort study was conducted at UPMC Children’s Hospital of Pittsburgh after approval by the University of Pittsburgh Institutional Review Board (IRB Study #STUDY22120083; approved on 17 May 2024). Medical records were reviewed for all infants admitted to the neonatal intensive care unit (NICU) who underwent surgical procedures and had documentation of epidural catheter placement in the chart between January 2018 and December 2024. No weight threshold was applied at the time of screening, and all medical records were reviewed and included for all infants that met both criteria. The study was conducted in accordance with the principles of the Declaration of Helsinki. The requirement for informed consent was waived due to the retrospective study design, and only de-identified data were collected.

### 2.2. Epidural Catheter Placement

The infants with no contraindications for epidural analgesia, were scheduled to receive either a caudal-threaded epidural catheter or a directly placed lumbar or thoracic epidural catheter. The choice of epidural technique and infusion medication was based on the attending pediatric regional anesthesiologist’s clinical judgment and preference. All epidural catheters were placed and managed by the Pediatric Acute Pain Service. Ultrasound guidance was used to identify the caudal or epidural space, guide needle placement, monitor catheter advancement when feasible, and/or confirm epidural catheter tip location. No additional imaging modalities or confirmation techniques, including fluoroscopy or radiography, were used. Caudal catheters were tunneled per the attending’s preference; however, this information was not reliably documented in the patient’s chart and was not collected. Direct epidural catheters were not tunneled. Medication for epidural infusions (ropivacaine with or without 1 mcg/mL clonidine), including programmed intermittent bolus (PIB) administration when prescribed, was individualized based on each patient’s age and weight, not to exceed 0.5 mg/kg/hour of ropivacaine for infants less than 4 months and 0.25–0.3 mg/kg/hour for neonates.

### 2.3. Data Collection

Data were collected retrospectively from the electronic medical record and included demographic and perioperative variables. Demographic variables included gestational age at birth, sex, postnatal age at surgery, corrected gestational age at surgery, and weight at surgery.

Intraoperative variables included American Society of Anesthesiologists (ASA) physical status, surgical procedure, anesthesia and surgical duration, epidural documentation of placement technique (caudal-threaded or direct lumbar/thoracic) and its success, vertebral level of needle insertion, epidural catheter tip location, time required for epidural catheter placement, intraoperative complications from epidural placement, and analgesic administration. Technical success was defined as the successful advancement of an epidural catheter to the desired vertebral level and the administration of ropivacaine. Placement failure was defined as abandonment of the procedure due to inability to access the epidural space, inability to advance the catheter to the desired level, suspected intrathecal or intravascular catheter placement, or needle and catheter resistance during placement. An attempt was defined as the insertion of a needle or the advancement of a catheter. A patient could have one successful catheter placement, one or two failed catheter placements when both caudal and direct epidural techniques were used, and multiple needle and catheter passage attempts.

Epidural analgesia variables include the administered local anesthetic and its concentration, use of clonidine, epidural infusion rates, total ropivacaine dose (mg/kg), use of programmed intermittent bolus (PIB), duration of epidural infusion, postoperative day (POD) of epidural catheter removal, and the documented reason for catheter removal.

Postoperative variables included pain and sedation scores assessed every 1–4 h (every hour immediately after surgery) using the Neonatal Pain, Agitation, and Sedation Scale (N-PASS) from POD 0 through POD 5; postoperative daily and total opioid consumption; administration of non-opioid analgesic medications; and catheter-related complications. Medication administration was recorded in 24-h intervals until epidural catheter removal. Intraoperative analgesics included fentanyl, morphine, methadone, and acetaminophen. Postoperative medications included morphine, fentanyl, and acetaminophen. All intraoperative and postoperative opioid doses were converted to intravenous morphine milligram equivalents (MMEs; mg/kg) for analysis.

Procedure-related complications were identified from the anesthesia and Pediatric Acute Pain Service consults. Intraoperative complications included intravascular catheter placement and suspected intrathecal catheter placement. Postoperative complications included catheter leakage, accidental catheter disconnection, catheter dislodgement, and other catheter-related events requiring premature catheter removal.

### 2.4. Study Outcomes

The primary objective of this retrospective observational cohort study was to describe the technical success of epidural catheter placement. The secondary objective was to compare postoperative analgesic outcomes between the two epidural techniques by evaluating longitudinal postoperative pain and sedation scores and postoperative opioid consumption.

The third objective was to compare the incidence of intraoperative and postoperative complications associated with each technique.

Unadjusted exploratory analyses were performed to evaluate the associations of epidural catheter technique, programmed intermittent bolus (PIB) administration, and epidural medications with postoperative opioid consumption and postoperative complications.

### 2.5. Statistical Analysis

Continuous variables are presented as median and interquartile range (IQR), and categorical variables as counts and percentages. Continuous variables were compared using the Wilcoxon rank-sum test, and categorical variables were compared using the chi-square test or Fisher’s exact test, as appropriate. Data distributions were evaluated using histograms, and correlations among continuous variables were assessed using correlation matrices.

Linear mixed-effects models were used to compare postoperative pain and sedation scores between the caudal-threaded and direct lumbar/thoracic epidural groups over POD 0–5. Because N-PASS assessments were documented clinically every 1–4 h, repeated assessments were first aggregated within each patient and postoperative day; the unit of observation in the models was therefore the patient-day, defined as the mean of all N-PASS scores recorded for that patient on that POD. Fixed effects included treatment group and POD, with a random intercept for each patient to account for repeated patient-day measurements. Interaction effects between treatment group and postoperative day were evaluated.

Exploratory univariate linear regression analyses were performed to evaluate the associations between epidural catheter technique, PIB administration, total epidural clonidine dose, and epidural ropivacaine dose and total postoperative opioid consumption, expressed as intravenous morphine milligram equivalents (MME/kg). Total postoperative MME/kg values were log-transformed before analysis because postoperative opioid consumption was right-skewed. Separate univariate models were fit for each exposure. Given the limited number of outcome events, multivariable adjustment was not statistically feasible. All regression analyses were therefore univariate and unadjusted, and are reported as exploratory and hypothesis-generating rather than as estimates of independent effect. Logistic regression models were fit to test the same exposures against binary outcomes of post op complications.

Results are reported as estimated mean differences with 95% confidence intervals (CI). Missing observations were excluded from the corresponding analyses. No formal a priori sample-size calculation was performed; all eligible cases during the study period were included, and the precision of the available sample is reflected in the reported 95% confidence intervals. All statistical tests were two-sided, and a *p*-value < 0.05 was considered statistically significant. Statistical analyses were performed using R version 4.3.1 (R Foundation for Statistical Computing, Vienna, Austria).

## 3. Results

A total of 104 infants underwent 105 epidural catheter placements by the Pediatric Acute Pain Service.

### 3.1. Epidural Catheter Technique Placement Failure

Five epidural catheter placements (two caudal-threaded and three direct lumbar/thoracic epidurals) failed in four infants, as described below.

One infant, 12 weeks (gestational age 25 weeks, 3.3 kg), had a failed caudal-threaded epidural placement because the catheter could not be advanced, and very high resistance was felt. During the second attempt, aspiration of clear fluid raised concern for intrathecal catheter placement, prompting termination of the procedure.

In a second patient, 8 weeks (gestational age 29 weeks, 3.53 kg), both caudal-threaded (three attempts) and direct lumbar/thoracic (T12–L1) epidural catheter placement (two attempts) failed because the catheter could not be advanced beyond the T12–L1 vertebral space. When advancing the catheter, very high resistance was encountered with each attempt.

In the third infant, 8 weeks (gestational age 26 weeks, 2.7 kg), direct lumbar/thoracic (T12–L1) epidural catheter placement was abandoned after two intravascular catheter placements were diagnosed after blood aspiration and an increase in heart rate and T waves of more than 25% from baseline with 0.27 mL of lidocaine 1.5% with epinephrine 1/200,000 injected via the epidural catheter.

In the fourth patient, 24 weeks (gestational age 35 weeks, 7.3 kg), direct thoracic (T8–T9, T9–T10) epidural catheter placement was aborted after five failed attempts to access the epidural space. None of these patients developed any complications from the multiple attempts performed during failed epidural placement.

### 3.2. Epidural Catheter Technique: Successful Placement

A total of 100 out of 105 epidural catheters (95.23%) were successfully placed in 100 infants (patient success rate: 96.15%). A total of 70 out of 72 infants (97.22%) received caudal-threaded epidural catheters, and 30 out of 33 infants (90.0%) received a direct epidural catheter placement, with lumbar insertion levels ranging from L4 to L1 (n = 8) and thoracic insertion levels ranging from T12 to T7 (n = 22). Epidural catheter tips were positioned between T4 and T12 under ultrasound guidance.

### 3.3. Patient Demographics, Surgical and Epidural Characteristics

Patient demographics, surgical and epidural characteristics are summarized in Table 1. Among the 100 infants, the median gestational age at birth was 34.5 weeks (IQR, 26.4–37.6). At the time of surgery, the median postnatal age was 7.9 weeks (IQR, 0.7–13.1), and the median corrected gestational age was 40.0 weeks (IQR, 38.4–42.1). Median weight at surgery was 3.0 kg (IQR, 2.52–3.42). The infants who received a direct lumbar/thoracic epidural catheter had a slightly higher corrected gestational age at the time of surgery (*p* = 0.045). Laparotomy with bowel resection was the most common procedure in the caudal-threaded epidural group. In contrast, jejunostomy, ileostomy, and colostomy procedures were proportionally more frequent in the direct lumbar/thoracic epidural group (Table 1).

### 3.4. Epidural Medication Administration

There was no significant difference between groups in the total intraoperative ropivacaine dose administered. The postoperative epidural infusion consisted of ropivacaine at concentrations of 0.05% (n = 1), 0.1% (n = 90), or 0.2% (n = 9), with clonidine (1 μg/mL) added in 17 patients. Ropivacaine 0.1% was used more often in the caudal-threaded epidural group (96% vs. 77%), whereas ropivacaine 0.2% was used more frequently in the direct lumbar/thoracic epidural group (23% vs. 3%) (Table 2).

More patients in the direct lumbar/thoracic epidural group received ropivacaine with clonidine (14 [47%]) than in the caudal-threaded group (3 [4%]; *p* < 0.001) as per provider’s preference. However, there was no statistical difference in total clonidine administered between the groups. Programmed intermittent bolus (PIB) administration was used in 20 patients at the provider’s discretion. Patients in the direct lumbar/thoracic epidural group were significantly more likely to receive PIB than those in the caudal-threaded group (60% vs. 3%; *p* < 0.001). Patients who received a direct lumbar/thoracic epidural catheter had a lower ropivacaine infusion on POD 3 (*p* = 0.006) and, overall, received higher cumulative postoperative ropivacaine doses. However, the difference was not statistically significant (*p* = 0.058) (Table 2).

### 3.5. Postoperative Pain Scores

**Figure 1:**
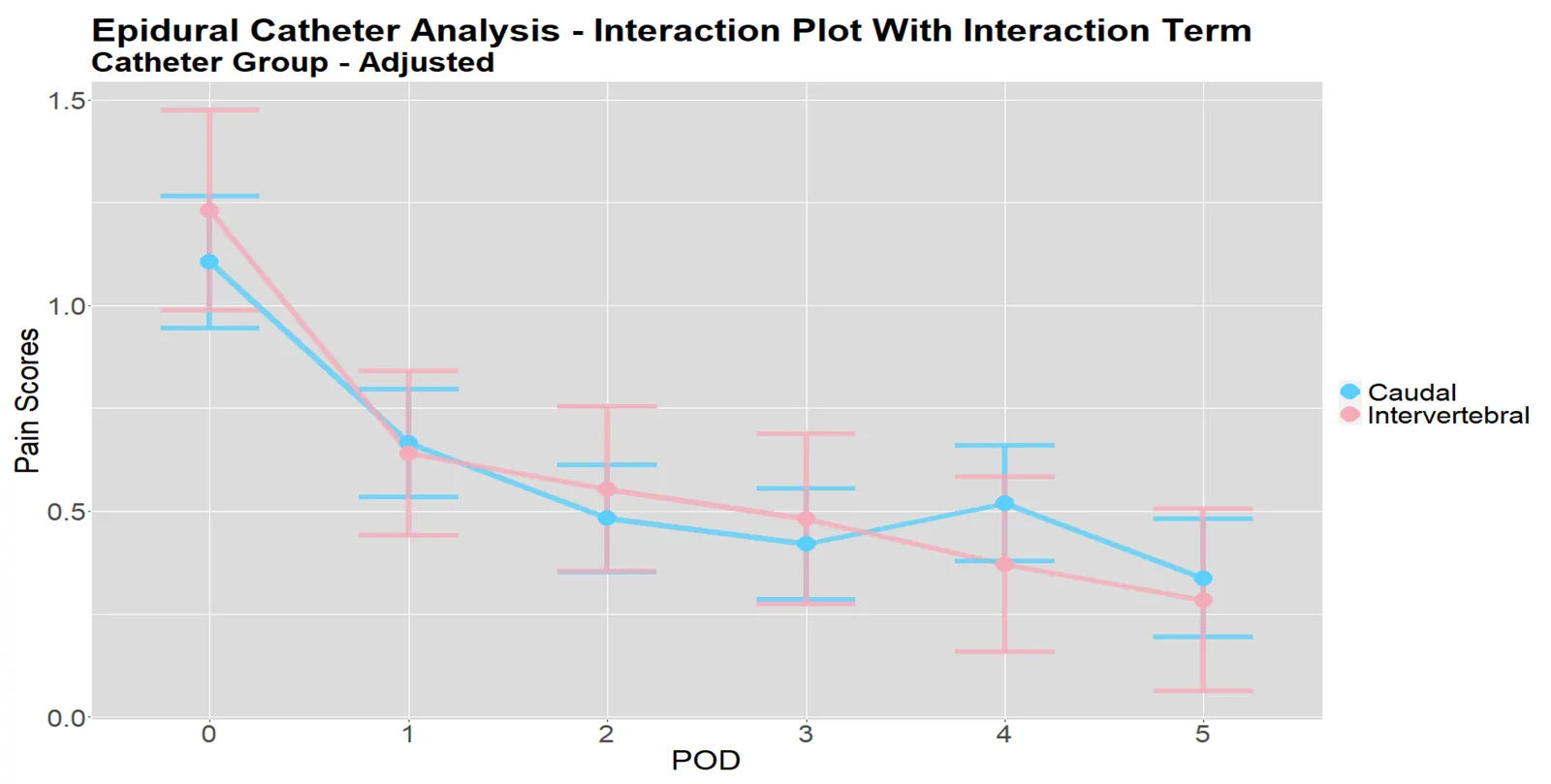
Adjusted daily mean postoperative pain scores over postoperative days (POD) 0–5 estimated from the linear mixed-effects model for 100 infants. The “Caudal” group shown in the figure corresponds to the caudal-threaded epidural catheter group, and the “Intervertebral” group corresponds to the direct lumbar/thoracic epidural catheter group. Points represent the adjusted mean pain scores, and error bars indicate the 95% confidence intervals. Pain scores decreased significantly over time (*p* < 0.001), with no significant difference between epidural catheter techniques (*p* = 0.972). POD is postoperative day.

There was no significant difference in mean postoperative pain scores between the caudal-threaded and direct lumbar/thoracic epidural groups (estimated mean difference, 0.00; 95% CI, −0.21 to 0.22; *p* = 0.972). Overall, postoperative pain scores decreased significantly over time (*p* < 0.001), with significantly lower N-PASS scores observed on each postoperative day compared with POD 0 (Figure 1).

### 3.6. Postoperative Sedation Scores

**Figure 2:**
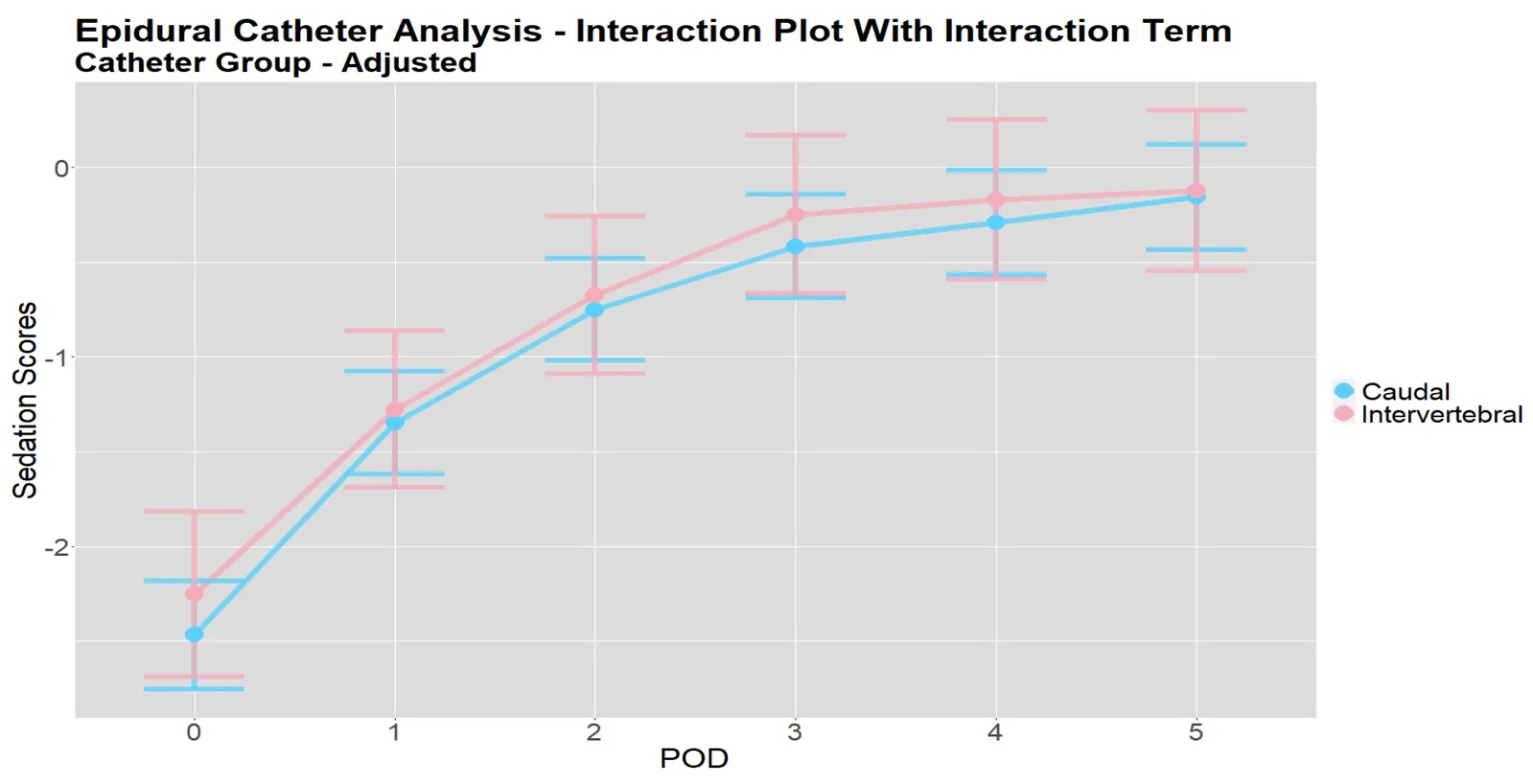
Adjusted mean daily postoperative sedation scores over postoperative days (POD) 0–5 estimated from the linear mixed-effects model in 100 infants. In the figure, “Caudal” refers to the caudally threaded epidural catheter group and “Intervertebral” refers to the direct lumbar/thoracic epidural catheter group. Points represent the adjusted mean sedation scores, and error bars indicate the 95% confidence intervals. Sedation scores increased significantly over time (became less negative), indicating decreasing levels of sedation during postoperative recovery (*p* < 0.001), with no significant difference between the two epidural catheter techniques (*p* = 0.673).

There was no significant difference in sedation scores between the caudal-threaded and direct lumbar/thoracic epidural groups (estimated mean difference, 0.10; 95% CI, −0.38 to 0.59; *p* = 0.673). Sedation scores changed significantly over time (*p* < 0.001), with progressively higher N-PASS sedation scores on each postoperative day compared with POD 0, indicating decreasing sedation levels during postoperative recovery (Figure 2).

### 3.7. Perioperative Analgesics Consumption

There was no difference in the intravenous MME (mg/kg) administered intraoperatively and postoperatively between the groups (*p* = 0.199). However, more patients in the caudal-threaded group, 62 (92%) vs. 21 (70%), received acetaminophen postoperatively (*p* = 0.019).

In unadjusted univariate analysis, epidural catheter technique was not associated with postoperative opioid consumption (estimate, −0.06; 95% CI, −0.77 to 0.64; *p* = 0.857). Similarly, neither PIB administration (estimate, −0.06; 95% CI, −0.87 to 0.76; *p* = 0.889) nor total epidural clonidine dose (estimate, 0.08; 95% CI, −0.01 to 0.17; *p* = 0.081) was associated with postoperative opioid consumption. In contrast, in unadjusted univariate analysis, higher cumulative epidural ropivacaine doses, including the combined intraoperative and postoperative dose (estimate, 0.08; 95% CI, 0.04 to 0.13; *p* = 0.001) and the postoperative dose alone (estimate, 0.02; 95% CI, 0.01 to 0.03; *p* = 0.001), were associated with greater postoperative opioid consumption. The intraoperative epidural ropivacaine bolus dose was not associated with postoperative opioid consumption (estimate, 0.05; 95% CI, −0.06 to 0.16; *p* = 0.370).

### 3.8. Perioperative Epidural Catheter Complications

During epidural placement, in the caudal-threaded epidural group, one patient experienced intravascular needle placement, and two patients had intravascular catheter placement; all were immediately recognized, and the epidural catheters were successfully repositioned. None of these patients developed any complications from the intravascular catheter placement.

Postoperative catheter-related complications occurred more frequently in the direct lumbar/thoracic epidural group than in the caudal-threaded epidural group (OR 5.58, 95% CI 1.29–24.09; *p* = 0.021). In the caudal-threaded group, complications included catheter leakage (n = 2) and ecchymosis (n = 1); none required premature catheter removal. In the direct lumbar/thoracic epidural group, complications included catheter leakage (n = 5) and accidental catheter disconnection (n = 1), all of which resulted in premature catheter removal. In unadjusted univariate logistic regression, patients who did not receive PIB had lower odds of postoperative catheter-related complications than those who did (OR, 0.09; 95% CI, 0.02–0.41; *p* = 0.002). Catheter leakage occurred in 6 of 20 patients (30%) who received PIB compared with 1 of 80 patients (1.3%) who did not receive PIB. However, because PIB administration was strongly associated with catheter approach, the observed association between PIB use and catheter leakage cannot be interpreted as an independent effect of PIB.

## 4. Discussion

This retrospective observational cohort study describes a seven-year institutional experience with continuous epidural analgesia in infants with a median weight of 3 kg. It compares caudal-threaded and direct lumbar/thoracic epidural catheter techniques. Patients undergoing direct lumbar/thoracic epidural placement were slightly older, with greater corrected gestational age, and had longer procedure times than those receiving caudal-threaded catheters.

Despite the technical challenges associated with both approaches, failed epidural placement was uncommon. Five epidural catheter placement procedures were aborted due to inability to advance the catheter, suspected intrathecal placement, repeated intravascular placement, or inability to access the epidural space. In addition, several intravascular catheter placements were recognized immediately and successfully replaced without reported complications. These findings emphasize the importance of meticulous technique, ultrasound guidance, and experienced practitioners when performing epidural catheter placement in this vulnerable population.

Nevertheless, successful epidural catheter placement was achieved in 96.15% of infants undergoing either technique. However, the success rate may differ between caudal-threaded and direct lumbar/thoracic epidural placement. Previous studies have shown that successful advancement of a caudally placed epidural catheter depends on patient age and size. For example, Bösenberg et al. reported a 95% success rate in infants aged 4 weeks to 5 months (2.7–6.5 kg), with success declining in older children as lumbar lordosis develops [11]. Similarly, Tsui et al. reported an overall technical success rate of 98.2% when electrical stimulation guidance was used; however, in five patients (aged 5 months to 1.6 years), the catheter could not be advanced to the desired thoracic or lumbar level, and the procedure was abandoned [2]. In contrast, Valairucha et al. reported successful advancement of caudally placed epidural catheters to the lumbar or thoracic epidural space in only 68% of infants younger than six months, as confirmed radiographically [16].

Our successful caudal cohort consisted of young infants (median corrected gestational age was 39.8 weeks), with a median postnatal age of 7.4 weeks and a median weight of 3 kg. The advancement of the caudally placed epidural catheter failed in 2 of 72 infants with characteristics in which successful caudal catheter advancement is most common. Unfortunately, one of these two patients also could not have the epidural catheter placed at the T12-L1 intervertebral level. These findings highlight that caudal epidural catheter advancement from the sacral hiatus to a thoracic vertebral level can remain technically challenging even in small-weight infants, despite the use of ultrasound to assist with placement. Resistance encountered during catheter advancement should prompt reassessment rather than forceful advancement, as it may indicate catheter contact with a nerve root, dura, or another anatomic obstruction.

Both direct lumbar and thoracic epidural techniques have their own technical challenges. Direct lumbar epidural catheter placement avoids the need to advance the catheter over multiple vertebral levels and may reduce the risk of malposition associated with the caudal approach. However, in a study by Baidya et al., advancement of an epidural catheter from the lumbar interspace to the thoracic level was successful in only 5 of 40 patients, with 4 of the 5 patients being infants [17]. Similarly, Blanco et al. reported successful advancement from the L4–L5 interspace to the T12 level in only 17% of patients aged 0 to 96 months [18].

The direct thoracic epidural approach offers the theoretical advantage of more precise catheter placement, potentially reducing catheter migration and allowing lower volumes of local anesthetic to achieve adequate segmental analgesia. However, this technique carries potential risks, including vascular puncture, dural puncture, spinal cord injury, intravascular catheter placement, and inadvertent subarachnoid catheter placement [19]. Although these complications are uncommon, their consequences in small infants may be particularly serious.

Our successful direct epidural cohort consisted of young infants (median corrected gestational age 40.8 weeks), with a median postnatal age of 9.4 weeks and a median weight of 3.2 kg. In the present study, the placement of the direct epidural catheter failed in 3 of 33 patients. It occurred at both lumbar and thoracic levels and across a broad age range (8–24 weeks) and weight range (2.7–7.3 kg), due to difficulty advancing the catheter, intravascular placement, or inability to access the epidural space. Notably, in our study, direct thoracic placement could not be done even in the largest infant (7.3 kg), suggesting that both insertion level and patient size may be contributing factors.

An important finding of our study was that no statistically significant between-group differences in postoperative analgesic outcomes were detected between the two techniques. Pain scores remained low throughout the postoperative period, and postoperative opioid requirements did not differ significantly between groups. These findings suggest that, when successfully placed, both caudal-threaded and direct lumbar/thoracic epidural catheters can provide adequate postoperative analgesia in small-for-weight infants.

Large pediatric registries have shown that neuraxial regional anesthesia is associated with a very low rate of serious complications. The French-Language Society of Pediatric Anesthesiologists and the United Kingdom pediatric regional anesthesia audit both reported serious incident rates below 0.1% [8,10]. Similarly, the Pediatric Regional Anesthesia Network (PRAN) reported extremely low rates of severe neurologic injury and local anesthetic systemic toxicity across more than 100,000 regional anesthetic procedures, with most severe local anesthetic toxicity events occurring after intravascular bolus administration in infants younger than six months [3,9].

Consistent with these reports, no neurologic complications or local anesthetic systemic toxicity were observed in our cohort. The most common adverse events were catheter-related complications. The incidence of postoperative catheter-related complications differed between techniques. However, the confidence interval is very wide (1.29–24.09), possibly due to a small infant cohort and few complications, so the results should be interpreted with caution. Catheter leakage occurred more frequently after direct lumbar/thoracic epidural placement and was the reason for premature catheter removal. The leakage cannot be attributed solely to the epidural catheter insertion site. An important observation was that all infants in the direct lumbar/thoracic group that leaked also received PIB administration. In addition, based on our clinical experience, caudal-threaded catheters have a longer length within the epidural space. They are not always tunneled, whereas direct lumbar/thoracic epidural catheters are generally not tunneled. However, these details were not consistently documented in the medical records and therefore could not be included in our analysis. Future prospective studies should evaluate the relationship between epidural infusion technique, catheter length in the epidural space, and the presence of tunneling and catheter-related complications.

This study has several limitations. Its retrospective design introduces the possibility of selection and information bias. The choice of epidural technique was determined by clinician preference rather than randomization, and differences in patient characteristics and surgical procedures may have influenced outcomes. The relatively small sample size, particularly in the direct lumbar/thoracic epidural group, and the low number of catheter-related complications warrant caution when interpreting the observed differences in complication rates. Because only seven catheter-related complications occurred, multivariable adjustment for potential confounders including age, corrected gestational age, weight, surgical procedure, catheter insertion level, local anesthetic concentration, clonidine use, programmed intermittent bolus administration, acetaminophen use, and duration of epidural infusion was not feasible. All comparisons are therefore unadjusted and exploratory, and residual confounding cannot be excluded. Catheter tunneling and fixation technique were inconsistently documented and could not be evaluated. This prevented analysis of their potential impact on catheter-related complications. In addition, postoperative epidural analgesic management was not fully standardized, as PIB administration varied substantially between groups and may have influenced catheter leakage and other postoperative outcomes.

Despite these limitations, this study included all NICU epidural catheters managed by a dedicated Pediatric Acute Pain Service over 7 years. It provided a comprehensive description of epidural technique success, patient selection, procedural characteristics, postoperative analgesic outcomes, and complications associated with caudal-threaded and direct lumbar/thoracic epidural techniques. It showed that both techniques can be successfully placed in infants with a median weight of 3 kg, with no statistically significant between-group differences detected in postoperative analgesic outcomes. The primary distinction between the techniques was a higher incidence of postoperative catheter-related complications after direct lumbar/thoracic epidural placement. Prospective multicenter studies are needed to determine whether catheter placement technique or postoperative local anesthetic infusion strategies influence catheter-related complications in this population.

## 5. Conclusions

In this single-center retrospective observational cohort study, the success rates for caudal-threaded and direct lumbar/thoracic epidural catheter placement were high. No statistically significant between-group differences in postoperative analgesic outcomes were detected among infants admitted to the NICU. Direct lumbar/thoracic epidural catheter technique was associated with a higher incidence of catheter leakage, necessitating premature catheter removal. The complications cannot be attributed to catheter placement location alone. Because postoperative epidural management differed in the use of programmed intermittent bolus administration, the observed differences in complications between groups should be interpreted with caution. Prospective multicenter studies with standardized epidural management protocols are needed to define the optimal epidural catheter technique better and identify modifiable factors associated with catheter-related complications in this population.

## Figures and Tables

**Table 1:** The demographics of the patient population, surgical and epidural characteristics. Data are presented as median and interquartile range (IQR). Categorical variables are presented as n (%) calculated within each treatment group. POD is postoperative day. Thoracic procedures included tracheoesophageal fistula repair and congenital diaphragmatic hernia repair. Laparoscopic procedures were performed for intestinal malrotation, Nissen procedure, and colostomy opening.

Variable	Caudal-Threaded Epidural n = 70 n (%)	Direct Lumbar/Thoracic Epidural n = 30 n (%)	*p*-Value
Patient Characteristics			
Male	40 (57%)	17 (57%)	0.964
Female	30 (43%)	13 (43%)	
Gestational Age at Birth (Weeks)	35.1 (27.6–38)	34.1 (26.1–37.3)	
Days of Life at Surgery	52 (4–88)	66 (7.5–108.5)	0.154
Postnatal Age at Surgery weeks)	7.4 (0.6–12.6)	9.4 (1.1–15.5)	0.154
Corrected Gestational Age at Surgery (weeks)	39.8 (38–41.4)	40.8 (39.4–44.8)	0.045
Weight (kg)	3 (2.5–3.2)	3.2 (2.6–3.8)	0.117
Surgical Characteristics			
Thoracic Procedure	11 (16%)	3 (10%)	0.073
Laparotomy with Bowel Resection	31 (44%)	10 (33%)	
Jejunostomy, Ileostomy, Colostomy Creation	24 (34%)	15 (50%)	
Laparoscopic Procedure	4 (6%)	2 (7%)	
Epidural Characteristics			
Time for Placement (minutes)	20 (15.2–26.8)	26 (22.2–30.8)	0.006
Postoperative Day of Epidural Removal	3 (3–3)	3 (2–4)	0.763

**Table 2:** Epidural medication characteristics. Data are presented as median and interquartile range (IQR). Categorical variables are presented as n (%) percentage calculated within each treatment group. POD is postoperative day. mg is milligrams, mcg is micrograms, kg is kilograms, h is hours.

Variable	Caudal-Threaded Epidural n = 70 n (%)	Direct Lumbar/Thoracic Epidural n = 30 n (%)	*p*-Value
Ropivacaine Infusion 0.05%	1 (1%)	0 (0%)	0.003
Ropivacaine Infusion 0.1%	67 (96%)	23 (77%)	
Ropivacaine Infusion 0.2%	2 (3%)	7 (23%)	
Epidural Infusion Duration (h)	63.8 (60.6–69)	63.7 (47.1–84.6)	0.946
Ropivacaine Infusion 0.1% (POD 0)	0.7 (0.6–0.8)	0.7 (0.5–0.8)	0.810
Epidural Infusion Rate (mL/h) (POD 1)	0.7 (0.6–0.8)	0.7 (0.5–0.8)	0.831
Epidural Infusion Rate (mL/h) (POD 2)	0.7 (0.6–0.8)	0.6 (0.5–0.8)	0.237
Epidural Infusion Rate (mL/h) (POD 3)	0.7 (0.5–0.8)	0.4 (0–0.8)	0.006
Ropivacaine Postoperatively (mg)	46.4 (36.9–57.3)	56 (31.9–88)	0.058
Total Combined Ropivacaine Intraoperatively and Postoperatively (mg/kg)	17.4 (15.3–20)	18 (14.7–29.5)	0.336

## Data Availability

The data presented in this study are available on request from the corresponding author due to privacy reasons.
